# Nano-Sized Secondary Organic Aerosol of Diesel Engine Exhaust Origin Impairs Olfactory-Based Spatial Learning Performance in Preweaning Mice

**DOI:** 10.3390/nano5031147

**Published:** 2015-06-30

**Authors:** Tin-Tin Win-Shwe, Chaw Kyi-Tha-Thu, Yadanar Moe, Fumihiko Maekawa, Rie Yanagisawa, Akiko Furuyama, Shinji Tsukahara, Yuji Fujitani, Seishiro Hirano

**Affiliations:** 1 Center for Environmental Health Sciences, National Institute for Environmental Studies, 16-2 Onogawa, Tsukuba, Ibaraki 305-8506, Japan; E-Mails: fmaekawa@nies.go.jp (F.M.); yanagisawa.rie@nies.go.jp (R.Y.); 2 Division of Life Science, Graduate School of Science and Engineering, Saitama University, 255 Shimo-Okubo, Sakura-ku, Saitama 338-8570, Japan; E-Mails: chawchaw25@gmail.com (C.K.-T-T.); yadanarmoe.91@gmail.com (Y.M.); stsuka@mail.saitama-u.ac.jp (S.T.); 3 Center for Environmental Risk Research, National Institute for Environmental Studies, 16-2 Onogawa, Tsukuba, Ibaraki 305-8506, Japan; E-Mails: kawagoe@nies.go.jp (A.F.); fujitani.yuji@nies.go.jp (Y.F.); seishiro@nies.go.jp (S.H.)

**Keywords:** developmental neurotoxicity, diesel exhaust, secondary organic aerosol, olfactory-based learning, preweaning mice, hippocampus, nanotoxicity

## Abstract

The aims of our present study were to establish a novel olfactory-based spatial learning test and to examine the effects of exposure to nano-sized diesel exhaust-origin secondary organic aerosol (SOA), a model environmental pollutant, on the learning performance in preweaning mice. Pregnant BALB/c mice were exposed to clean air, diesel exhaust (DE), or DE-origin SOA (DE-SOA) from gestational day 14 to postnatal day (PND) 10 in exposure chambers. On PND 11, the preweaning mice were examined by the olfactory-based spatial learning test. After completion of the spatial learning test, the hippocampus from each mouse was removed and examined for the expressions of neurological and immunological markers using real-time RT-PCR. In the test phase of the study, the mice exposed to DE or DE-SOA took a longer time to reach the target as compared to the control mice. The expression levels of neurological markers such as the *N*-methyl-d-aspartate (NMDA) receptor subunits NR1 and NR2B, and of immunological markers such as TNF-α, COX2, and Iba1 were significantly increased in the hippocampi of the DE-SOA-exposed preweaning mice as compared to the control mice. Our results indicate that DE-SOA exposure *in utero* and in the neonatal period may affect the olfactory-based spatial learning behavior in preweaning mice by modulating the expressions of memory function–related pathway genes and inflammatory markers in the hippocampus.

## 1. Introduction

Exposure to environmental pollutants containing nano-sized particles during the developmental period might represent a major risk factor for children or the health of the next generation. Early diagnosis is necessary for proper treatment to prevent disability in the later life of children. A variety of species have the ability to learn from the surrounding cues and use such spatial memory to navigate, using landmarks from any position to a specific location. Many tests are well-established to examine learning and memory functions in adults, however, there are very limited tests to examine learning and memory functions in neonates. There is only one report in the literature indicating that olfactory-based spatial learning in neonatal mice is associated with Ca^2+^/calmodulin-dependent protein kinase (CaMKII) in the hippocampal neurons [1]. With this background, we were prompted to establish a novel neonatal mouse model for early examination of learning using olfactory-based learning performance.

Ambient particulate matter consists of primary particles emitted directly from sources, and secondary particles formed by chemical reactions between particles and gases in the atmosphere, which are known as secondary organic aerosols (SOAs). Nowadays, the importance of SOA formation in urban areas is well-recognized, not only in the atmosphere but also in indoor environments [2,3]. Previously, our laboratory has reported the effects of primary particles, such as carbon black nanoparticles and nanoparticle-rich diesel exhaust (DE), on the brain's inflammatory mediators, neurotransmitter system, memory function–related gene expressions, and learning performance in adult mice [4]. We have demonstrated the neuroinflammatory effects of exposure to carbon black nanoparticles in adult BALB/c mice [5], and the neurotoxic effects of exposure to carbon black nanoparticles, by measuring the excitatory amino acid neurotransmitter levels in the olfactory bulb [6]. Moreover, we have also shown the effects of nanoparticle-rich diesel exhaust exposure on the changes of brain neurotransmitters, inflammatory biomarkers, and learning ability in adult mice [7,8,9,10]. It has been reported that exposure to SOA emitted from coal-fired power plants may be associated with an increased risk of heart disease in susceptible animals [11]. Currently, we have established an SOA exposure system by mixing diesel exhaust with ozone to generate an SOA. Recently, we showed that SOA exposure impaired maternal behavior and novel object recognition ability in BALB/c mice [12]. Our findings suggest that gestational exposure to SOA affects neonatal mice via the transplacental route. These findings prompted us to examine the effect of exposure to SOAs and study the mechanism underlying the effects of SOAs in neonatal animals.

The purpose of the present study was to establish a preweaning mouse model for early detection of an olfactory-based spatial learning disability and for examining the effects of exposure to environmental pollutants during the developmental period at an early age. We hypothesized that the potential toxic substances contained in DE-origin SOA (DE-SOA) may reach the brain via the olfactory nerve route or via the systemic circulation to induce olfactory-based learning deficits in preweaning mice. This is the first study to report that exposure to SOA of diesel engine exhaust origin in the developmental stage affects olfactory-based learning performance and the related gene expressions in the brain of preweaning mice.

## 2. Results

### 2.1. Effects of DE or DE-SOA on Olfactory-Based Spatial Learning Ability

**Figure 1:**
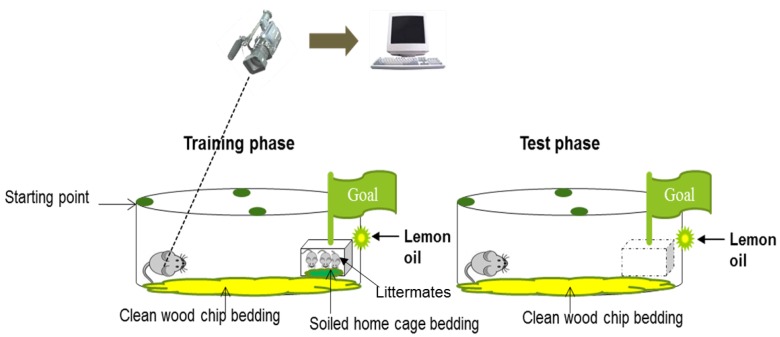
Establishment of the olfactory-based spatial learning test for preweaning mice.

**Figure 2:**
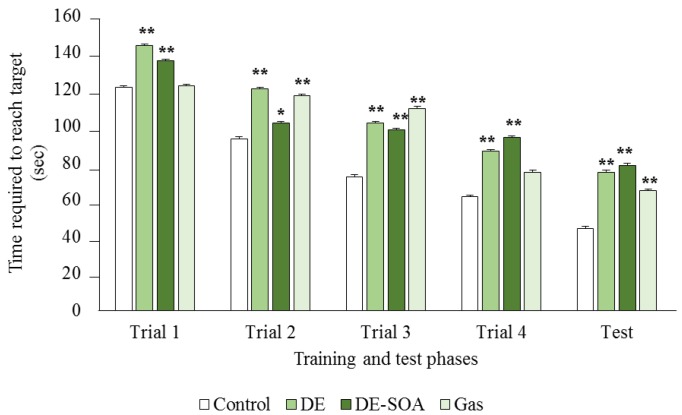
Effects of diesel exhaust (DE) or DE-SOA (secondary organic aerosols) on olfactory-based spatial learning ability in preweaning mice. (*n* = 8–10, ** *p* < 0.01, * *p* < 0.05 *vs.* control).

First, we established the preweaning mouse model that could be used for early diagnosis of learning deficits after developmental exposure to environmental pollutants (Figure 1). We used preweaning male mice on postnatal day (PND) 11 for the spatial learning test, because mouse pups at that age have not yet opened their eyes and use only olfactory cues. We measured the time required for the mice to reach the target and found that the mice exposed to DE, DE-SOA, or gas took a longer time to reach the target as compared to the control mice (Figure 2).

### 2.2. Odor Discrimination and Motor Function Test

**Figure 3:**
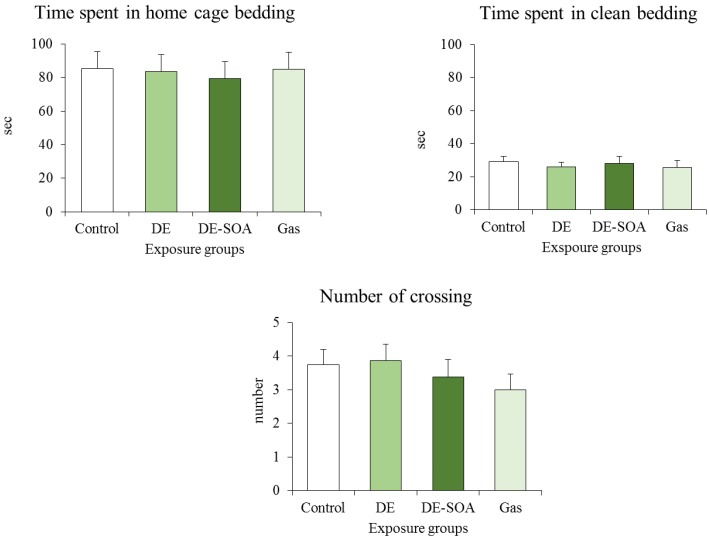
Odor discrimination and motor function test in PND 11 preweaning mice (*n* = 8).

In the present study, trial 1 in Figure 2 showed some evidence of impairment before the learning test, thus we performed an independent assessment of olfactory and motor deficits in a separate group (*n* = 8 for each group). We found that all pups from the control and exposure groups spent more time in the home cage bedding area compared to the clean bedding area within the groups (*p* < 0.001), and there was no difference of time spent in the home cage bedding area between the control and the exposure groups. We also found that locomotion, expressed as a number of crossing, was not different between groups (Figure 3).

### 2.3. Effects of DE and DE-SOA on NMDA Receptor Subunits and CaMKII Expressions in the Hippocampi of the Preweaning Mice

**Figure 4:**
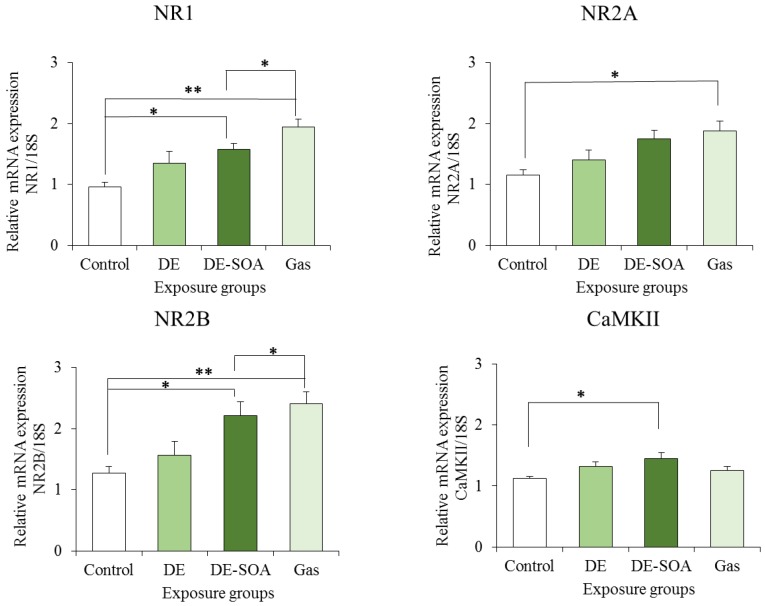
Effects of DE or DE-SOA exposure on the gene expressions of the NMDA receptor subunits NR1, NR2A, and NR2B, and of CaMKII in the hippocampi of the preweaning mice (*n* = 6–8, ** *p* < 0.01, * *p* < 0.05 *vs.* control).

The *N*-methyl-d-aspartate (NMDA)-type glutamate receptors in the hippocampus are essential for spatial learning and memory, as well as for the induction of synaptic plasticity [13,14]. It has been reported that olfactory-based spatial learning in neonatal mice is associated with CaMKII expression in the hippocampal neurons [1]. Thus, we examined the mRNA expression levels of the NMDA receptor subunits NR1, NR2A, and NR2B, and of the transcription factor CaMKII in the hippocampus, and found that the expression levels of NR1, NR2B, and CaMKII were significantly increased in the hippocampi of the DE-SOA-exposed preweaning mice as compared to the hippocampi of the control mice (Figure 4).

### 2.4. Effects of DE and DE-SOA on Inflammatory Marker Expressions in the Hippocampi of the Preweaning Mice

**Figure 5:**
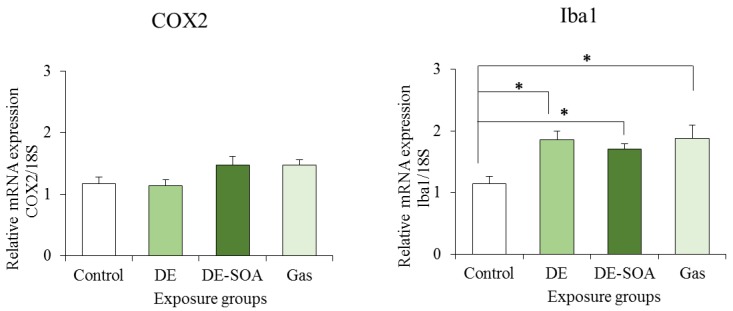
Effects of DE or DE-SOA on the expression of the inflammatory marker COX2 and microglial marker Iba1 in the hippocampi of the preweaning mice (*n* = 6–8, * *p* < 0.05 *vs.* control).

**Figure 6:**
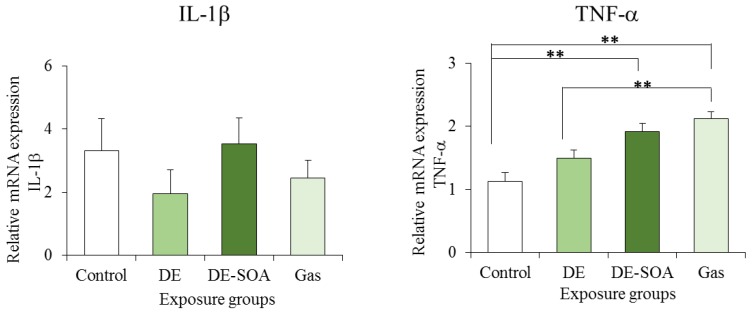
Effects of DE or DE-SOA on the expressions of the proinflammatory cytokine IL-1β and TNF-α in the hippocampi of the preweaning mice (*n* = 6–8, ** *p* < 0.01 *vs.* control).

Furthermore, the mRNA expression of Iba1, a microglial marker, and of the proinflammatory cytokine TNF-α, which is secreted from the microglia, were also increased significantly in the hippocampi of the preweaning mice exposed to DE-SOA when compared to the hippocampi of the control mice (Figure 5 and Figure 6). However, there were no significant differences in the mRNA expression levels of COX2 and IL-1β between the control and DE-SOA-exposed preweaning mice. Our results indicate that developmental exposure to SOA derived from diesel engine exhaust may affect olfactory-based spatial learning behavior in preweaning mice by modulating the expressions of the NMDA signaling pathway genes and inflammatory marker expressions in the hippocampus.

### 2.5. Effects of DE and DE-SOA on the Histology of the Hippocampus in the Preweaning Mice

**Figure 7:**
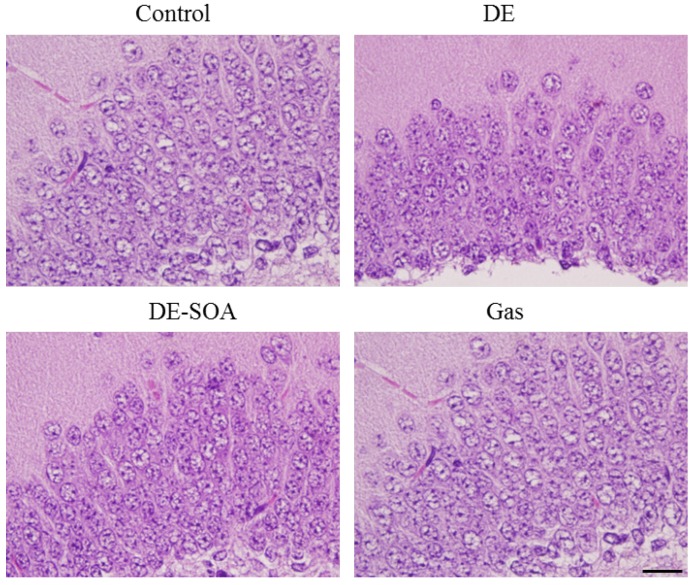
Representative photomicrographs showing the histology of CA1 area of the hippocampi in the control, DE-, DE-SOA-, and gas-exposed preweaning mice. Scale bar = 50 μm.

We investigated the histological changes of the hippocampi from the control, DE-, DE-SOA-, and gas-exposed mice using hematoxylin and eosin (H & E) staining. We did not observe any remarkable morphological differences between the control and the DE- or DE-SOA-exposed mice (Figure 7).

### 2.6. Correlation between Inflammatory Markers and NMDA Receptors

The Pearson correlation coefficient analysis was performed to detect the association between inflammatory marker TNF-α and NMDA receptor subunits (NR1, NR2A, NR2B). Correlation coefficient “*r*” values were expressed in Table 1. For TNF-α and NR1, a weak positive correlation was observed in the gas exposure group, a weak negative correlation was observed in the control group, and a moderate negative correlation was observed in the DE and DE-SOA exposure groups. For TNF-α and NR2A, a moderate positive correlation was observed in the control and gas exposure groups, and a strong positive correlation was observed in the DE and DE-SOA exposure groups. For TNF-α and NR2B, a moderate positive correlation was observed in the control and DE-SOA exposure groups, and a strong positive correlation was observed in the DE and gas exposure groups.

**Table 1:** Pearson coefficient value (*r*) showing association between TNF-a and NMDA

Receptor	NR1	NR2A	NR2B
Control	*r* = −0.385	*r* = 0.641	*r* = 0.623
	*p* = 0.346	*p* = 0.087	*p* = 0.099
DE	*r* = −0.630	*r* = 0.794	*r* = 0.927
	*p* = 0.050	*p* = 0.006	*p* = 0.000
DE-SOA	*r* = −0.732	*r* = 0.758	*r* = 0.638
	*p* = 0.016	*p* = 0.011	*p* = 0.047
Gas	*r* = 0.248	*r* = 0.745	*r* = 0.874
	*p* = 0.489	*p* = 0.013	*p* = 0.001

Notes: 0.1 < *r* < 0.3 means small correlation; 0.3 < *r* < 0.5 means moderate correlation; 0.5 < *r* < 1 means strong correlation.

## 3. Discussion

Epidemiological evidence has indicated the existence of positive associations between the inhalation of elevated levels of particulate matter (PM) and pulmonary and cardiovascular morbidities and mortality in susceptible populations [15,16,17,18]. DE is a major source of ambient PM in urban environments and one of the major precursors for SOA formation. SOAs are formed in the atmosphere by oxidation of products originating from anthropogenic and biogenic volatile organic compounds [19]. SOA formation may occur not only in the atmosphere, but also in indoor environments [2,3]. Previously, we have shown that a single intranasal administration of SOA induces inflammatory responses in the lungs by modulating the expressions of proinflammatory cytokines, transcription factors, and inflammation-responsive neurotrophins [20]. Recently, our research group reported that exposure to DE-SOA may affect the novel object recognition ability and impair the maternal behavior in adult mice [12]. In human studies, it has been reported from Mexico City that children with prefrontal lesions exposed to air pollution showed cognitive deficits [21,22]. An association has also been shown between air pollution and cognitive impairment in healthy individuals, including adult and elderly women [23,24]. In regard to DE, human studies have indicated that exposure to DE may affect the central nervous system [25,26]. An *in vitro* study indicated that decreased phagocytic activity was found in human macrophages exposed to SOA from alpha-pinene, and IL-8 production was increased in pig explants exposed to SOA from 1,3,5-trimethlbenzene with high particle numbers [27]. However, it is not clear whether an association may exist between exposure to SOA derived from DE and specific functions of the central nervous system, such as learning and memory functions.

In the present study, we first established a preweaning mouse model that could be used for early diagnosis of learning deficits after developmental exposure to environmental pollutants. Early diagnosis is necessary for proper treatment to prevent disability later in life in the children. Adult rodents learn spatial constellation from visual cues, but in preweaning mammals, olfactory cues play an important role in homing [28,29]. Many tests have been established to examine learning and memory functions in adults. However, there are very limited tests to examine learning and memory functions in neonates. After establishing a novel olfactory-based learning test, we investigated the effects of developmental exposure to SOA, a model environmental pollutant, on the spatial learning performance of preweaning mice, and we measured the memory function–related gene expressions and immune biomarker levels in the hippocampus. The major findings of the present study are that preweaning mice exposed to DE, DE-SOA, or gas without nanoparticles during the gestational and lactational periods showed poor olfactory-based spatial learning ability as compared to the controls. Moreover, the expression levels of the NMDA receptor subunits NR1 and NR2B, and of immunological markers such as TNF-α, were significantly increased in the hippocampi of the DE-SOA-exposed preweaning mice as compared to the hippocampi of the control mice.

Regarding the olfactory-based spatial learning ability in the present study, most of the pups, including control group, had a first experience and did not know how to learn and how to reach the target in the first trial. The examiner guided the pups that failed to reach the target and placed the pup at the target for 1 min to learn. In the second, third, and fourth trials and test phase, most of the pups could reach the target, although the time to reach the target is dependent on the exposure groups. If there are severe olfactory impairments and motor deficits from exposure, the pups are not supposed to reach the target after the learning experience by trials. The mild adverse effects on olfaction and the motor system should be excluded. We provided the following two results, indicating the possibility that the impairment of olfaction and the motor system did not occur by exposure: (1) we examined the level of mRNA expression of inflammatory cytokines and histopathology in the olfactory bulb and we did not find any significant changes between the control and exposure groups; (2) we performed an independent assessment of the odor discrimination and motor function test in PND 11 mouse pups in a separate group and we did not find olfactory impairment and motor deficit. Taken together, we suggest that spatial learning deficit in the present study was not due to olfactory and motor impairment.

For adult rodents, visual cues are important to learn about their place or home, for example, by the Barnes maze or the Morris water maze test [28,29]. However, in preweaning mammals whose eyes have not yet opened, olfactory cues are important for the navigation of the newborns to their mothers or littermates [30]. In the present study, we investigated the olfactory-based navigation to the target goal of PND 11 preweaning mice after gestational and lactational exposure to DE, DE-SOA, or gas without nanoparticles. We found that preweaning mice exposed to DE, DE-SOA, or gas during the brain development period showed an increased time to reach the target as compared to the control mice. NMDA-type glutamate receptors in the hippocampus are essential for spatial learning and memory, as well as for the induction of synaptic plasticity [13,14]. Knockout mice lacking the NR2 subunit of the NMDA receptor in CA1 have been demonstrated to show severely impaired performances in the Morris water maze test [13]. The poor olfactory-based spatial learning ability in the preweaning pups exposed to DE and DE-SOA was shown to be accompanied by significant increases in the expressions of NMDA receptor subunits such as NR1 and NR2B, of the microglial marker Iba1, and of the proinflammatory cytokine TNF-α. Therefore, we suggest that the potential toxic substances contained in DE, DE-SOA, or gas may reach the brain directly and cause abnormal activation of the NMDA receptor subunits and CaMKII in the hippocampus. It can be postulated that up-regulation of the NMDA receptor subunits NR1 and NR2B in animals exposed to DE-SOA during the brain development period may be due to changes in the regulation of the subunit expression, localization, and post-translational modifications and interactions with other receptors or regulatory proteins. Moreover, expression of the major immune cell microglial marker Iba1 was increased significantly in all three exposed groups as compared to the control group. Expressions of the proinflammatory cytokine TNF-α was also increased significantly in the DE-SOA- and gas-exposed group as compared to the control group. Microglia are the major source of cytokines during neuroinflammation. It is possible that DE or DE-SOA may induce glutamate release from hemichannels of gap junctions via TNF-α-TNFR1 signaling and rapidly increase the excitatory synaptic strength by inducing increased Ca^2+^ permeable-α-amino-3-hydroxy-5-methyl-isoxazole-4-propionic acid (AMPA) receptors and/or NMDA receptors [31,32] and may lead to neurotoxicity. Although the number of animals was small, we found a correlation between inflammatory marker TNF-α and NMDA receptor subunits in the present study. Further studies are needed to clarify the role of the neuroimmune network in the brain on olfactory-based learning ability after early life exposure to environmental pollutants. Taken together, we suggest that the potential substances contained in DE, DE-SOA, or gas may reach the brain via the olfactory nerve route or systemic circulation and induce inflammatory responses and spatial learning deficits.

CaMKII is a major neuronal mediator of calcium signaling and plays an important role in a variety of events in neurons, ranging from neurotransmitter synthesis and release, the modulation of neurotransmitter receptors and ionic channels, and gene expressions to several aspects of synaptic plasticity essential for learning and memory, called long-term potentiation (LTP), and spatial learning [33,34,35,36,37]. It has been reported that adult mice that show over-expression of CaMKII in the hippocampal neurons do not show LTP or impaired spatial learning ability in the Barnes maze [28,37]. Furthermore, it was shown that olfactory-based spatial learning in neonatal mice is associated with CaMKII expression in the hippocampal neurons [1]. In the present study, we found that the expression level of CaMKII mRNA in the hippocampus was significantly increased in the DE-SOA-exposed mouse pups that showed poor spatial learning ability as compared to the control pups. This suggests that CaMKII expressed in the hippocampus may be involved in olfactory-based spatial learning ability. However, no histological abnormalities were found in the hippocampi of any of the groups. Overall, we have established a novel olfactory-based spatial learning test for young rodents, which makes it possible to detect the effects of developmental exposure to environmental pollutants or toxins.

## 4. Materials and Methods

### 4.1. Animals

Pregnant BALB/c mice were purchased from Japan SLC Co. (Tokyo, Japan) and exposed to clean air, diesel exhaust (DE), DE-origin SOA (DE-SOA), or gas without particles (Gas) from gestational day 13 to postnatal day (PND) 10 (5 h/day for 5 days) in exposure chambers. Food (a commercial CE-2 diet, CLEA Japan, Inc., Tokyo, Japan) and water were given *ad libitum*. The male mice were housed in wire cages under controlled environmental conditions (temperature, 22 ± 0.5 °C; humidity, 50% ± 5%; lights on 07:00–19:00 h). For assessment of the olfactory-based learning behavior, we used PND 11 preweaning female mice, because mouse pups at that age have not yet opened their eyes, and they use olfactory cues only. There were two phases of the study, including the training phase (4 trials) and test phase (1 trial), and each trial lasted for 180 s. The time to reach the target goal cage which contained the littermates was recorded for each preweaning mouse using a video-assisted computer and software. This study was conducted with the approval of the Ethics Committee of the Animal Care and Experimentation Council of the National Institute for Environmental Studies (NIES), Tsukuba, Japan.

### 4.2. Generation of SOA

DE-SOA was generated at the National Institute for Environmental Studies, Japan. An 81-model diesel engine (J08C; Hino Motors Ltd., Hino, Japan) was used to generate the diesel exhaust. The details of the exposure system have been described previously [12,38]. The engine was operated under a steady-state condition for 5 h per day. In the present study, our driving condition of the diesel engine was not a simulation of any special condition, as it is in the real world. The engine operating condition (2000 rpm engine speed and 0 nm engine torque) in this study permitted a high concentration of nano-sized particles. There were four chambers in the system: a control chamber receiving clean air filtered using a HEPA filter and a charcoal filter (referred to as “clean air”); a chamber containing the diluted exhaust (DE, which was without mixing O_3_); a chamber containing DE-SOA, which was generated by mixing DE with O_3_ at 0.6 ppm after secondary dilution; and a chamber with gas only without particles. In the gas group, almost all the particles were filtered to get gaseous compounds only for excluding the fact that the effects were due to diesel exhaust origin particles and not due to gaseous compounds. For gas delivery, we used an ultra-low particulate air (ULPA) filter which is 99.999% able to trap a vast majority of very small particulate matter. The concentration of nanoparticles was 1.46 μg/m^3^ in the control chamber and 1.13 μg/m^3^ in the gas chamber. The size of the nanoparticles in both chambers was not measurable. Gaseous compounds in the four chambers were expressed in our previous study [12]. The secondary dilution ratio in the DE and DE-SOA chambers was the same, which resulted in the same particle and gaseous concentrations when O_3_ was not mixed. Actually, the concentrations of the particles in DE-SOA was higher when O_3_ was mixed and the concentrations of DE and DE-SOA were 110.83 ± 25.11 μg/m^3^ and 122.76 ± 24.54 μg/m^3^, respectively. The increased mass concentration was due to the secondarily generated particles. The difference between DE and DE-SOA was the ratio of water soluble organic carbon (WSOC) and organic carbon (OC). The WSOC/OC ratio was 0.11 in the DE chamber and 0.16 in the DE-SOA chamber. The temperature and relative humidity inside each chamber were adjusted to approximately 22 ± 0.5 °C and 50% ± 5%, respectively. The particle characteristics were evaluated from the air samples taken from inside the exposure chamber. In detail, sample air was taken from the breeding space of the inhalation chamber (2.25 m^3^) using stainless steel tubing. The gas concentrations (CO, CO_2_, NO, NO_2_, and SO_2_) were monitored using a gas analyzer (Horiba, Kyoto, Japan). CO and NO*_x_* concentrations in both chambers were similar, however, those of NO and NO_2_ differed from each other because NO was oxidized to NO_2_ by reaction with O_3_. The particle size distributions were measured using a scanning mobility particle sizer (SMPS 3034; TSI, Shoreview, MN, USA). The sizes of the particles used in the present study were 25.56 ± 1.75 nm for DE and 25.46 ± 1.51 nm for DE-SOA. The particles were collected using a Teflon filter (FP-500; Sumitomo Electric, Osaka, Japan) and a Quartz fiber filter (2500 QAT-UP; Pall, Pine Bush, NY, USA), and the particle mass concentrations were measured using a Teflon filter. The particle weights were measured using an electrical microbalance (UMX 2, Mettler-Toledo, Columbus, OH, USA; readability 0.1 μg) in an air-conditioned chamber (CHAM-1000; Horiba) under constant temperature and relative humidity conditions (21.5 °C, 35%). For the Quartz fiber filter, the quantities of elemental carbon and organic carbon were determined using a carbon analyzer (Desert Research Institute, Reno, NV, USA). An analysis of the particle composition (DE and DE-SOA) showed that the percentage of OC relative to the total carbon in the diluted exhaust was about 60% and the DE and DE-SOA showed nearly the same carbon composition.

### 4.3. Experimental Schedule

The preweaning mice were allocated to one of four different groups (*n* = 8 per group), as follows: (1) mice exposed to clean filtered air; (2) mice exposed to DE; (3) mice exposed to DE-SOA; and (4) mice exposed to gas without particles. The mice were exposed to clean air, DE, DE-SOA, or gas in a whole-body exposure chamber for 5 h per day (22:00–03:00 h) on 5 days of the week from GD 13 to PND 10 (Figure 1). On PND 11, the olfactory-based spatial learning ability of each mouse was examined. Immediately after the completion of the learning test, the mice were sacrificed and six of the eight mice were used for the mRNA analysis and two for the histological analysis.

### 4.4. Olfactory-Based Spatial Learning Test

We have established an olfactory-based spatial learning ability test based on [1], as presented in Figure 1. A rounded cage 40 cm in diameter with clean wood chip bedding was used as the test cage. The dam and four-to-five mouse pups, littermates of the test pup, were placed in a wire cage (target place) (8 × 10 × 5 cm) containing soiled home cage bedding. The rounded test cage was divided into four quadrants and this wire cage was placed in one quadrant. The start or release points were marked in the other three quadrants. Cotton impregnated with lemon oil was fixed on the wall behind the small wire cage to allow memorization identification of the target location. We used PND 11 preweaning male mice for the spatial learning test because mouse pups at that age have not yet opened their eyes and use olfactory cues only. There were two phases in the study, including the training phase (four trials) and the test phase (one trial) and each trial lasted for 180 s. The examiner guided the pups that failed to reach target and placed them at the target for 1 min to learn. The time to reach the target cage containing the littermates was recorded for each preweaning mouse using a video-assisted computer and software (Muromachi Kikai, Tokyo, Japan). In the training phase, four trials (180 s per trial) were given, with an interval between the trials of 5 to 15 min, and if the goal was not reached, the pup was guided at 180 s. After the fourth trial, and after the pup had spent 1 min in the huddle, the test phase (180 s) was carried out. In the test phase, to examine whether the test pups remembered the location of the target, the wire cage containing the dam and littermate mouse pups was removed from the big rounded test cage and the time to see if the test pups could reach the target was 180 s.

### 4.5. Odor Discrimination and the Motor Function Test

In the present study, trial 1 in Figure 2 showed some evidence of impairment before the learning test, thus we performed an independent assessment of olfactory and motor deficits in a separate group. Briefly, we used a different rectangular wire mesh cage (30 × 20 × 10 cm) placed on the tray, which was divided into three areas of same size (10 × 20 × 10 cm). In the tray, the home cage bedding was placed under the mesh at one side of the test cage, the clean bedding was placed under the mesh at the opposite side, and the center was kept blank as a neutral area. On the day of the odor discrimination and motor function test, PND 11 pups were taken out from the home cage and placed in the center. During two 3-min trials, the time spent by test pups in each of the three areas and the number of crossings between the areas was recorded.

### 4.6. Quantification of the mRNA Expression Levels

After completion of the olfactory-based spatial learning test, six-to-eight male mice from each group were sacrificed under deep sodium pentobarbital anesthesia and the hippocampi from all the mice were collected. Hippocampal samples were frozen quickly in liquid nitrogen then stored at –80 °C until extraction of the total RNA. Briefly, total RNA extraction from the samples was performed using the BioRobot EZ-1 and EZ-1 RNA tissue mini kits (Qiagen GmbH, Hilden, Germany). Then, the purity of the total RNA was examined, and the quantity was estimated using the ND-1000 NanoDrop RNA Assay protocol (NanoDrop, Wilmington, DE, USA), as described previously [5]. Next, we performed first-strand cDNA synthesis from the total RNA using SuperScript RNase H-H^−^Reverse Transcriptase II (Invitrogen, Carlsbad, CA, USA), according to the manufacturer’s protocol. Then, we examined the mRNA expressions of 18S, NR1, NR2A, NR2B, IL-1β, TNF-α, COX2, and Iba1 using a quantitative real-time RT-PCR method and the Applied Biosystems (ABI) Prism 7000 Sequence Detection System (Applied Biosystems Inc., Foster City, CA, USA). The tissue 18S rRNA level was used as an internal control. Some primers (NR1, NM_008169; NR2A, NM_008170; NR2B, NM_008171; IL-1β, NM_008361; COX2, NM_011198; Iba1, NM_019467) were purchased from Qiagen, Sample & Assay Technologies. 18S and TNF-α primers were designed and purchased from Hokkaido System Science. Data were analyzed using the comparative threshold cycle method. Then, the relative expression levels of the memory function–related genes and the related transduction pathway molecule mRNAs were individually normalized to the 18S rRNA content in the respective samples and expressed as mRNA signals per unit of 18S rRNA expression.

### 4.7. Histological Examination

The brains of two PND 11 preweaning mice each were removed from the control and DE, DE-SOA-, or gas-exposed groups after the animals had been deeply anesthetized with sodium pentobarbital; the brains were then fixed in 10% formalin. The fixed brains were dehydrated using a graded series of ethanol, cleared with xylene, and embedded in paraffin. Coronal paraffin sections were cut at a thickness of 5 μm using a microtome and were mounted on 3-aminopropyltriethoxysilane-coated glass slides. Each section was stained with hematoxylin and eosin (H&E) for histological examination.

### 4.8. Statistical Analysis

All the data are expressed as mean ± standard error (S.E.). The statistical analysis was performed using the StatMate II statistical analysis system for Microsoft Excel, Version 5.0 (Nankodo Inc., Tokyo, Japan). Messenger RNA data and odor discrimination and motor function test data were analyzed by one-way analysis of variance, with post-hoc analysis performed using the Bonferroni/Dunn method. Student’s *t* test was used to analyze the preference between home cage bedding and clean bedding. The Pearson correlation coefficient analysis was performed to detect the association between inflammatory marker TNF-α and NMDA receptor subunits (NR1, NR2A, NR2B). Significant level was *p* < 0.05.

## 5. Conclusions

Our results indicate that developmental exposure to DE-SOA may affect olfactory-based spatial learning behavior in preweaning mice by modulating NMDA receptors, signaling pathway gene CaMKII, and inflammatory markers in the hippocampus. We suggest that, although the potential toxic substances contained in DE-SOA have not yet been identified, they may reach the brain via the olfactory nerve route or systemic circulation and induce a spatial learning deficit. Further studies are needed to elucidate whether the persistency of the spatial learning deficit is present in adulthood or not.
